# The AIMS home-video method: parental experiences and appraisal for use in neonatal follow-up clinics

**DOI:** 10.1186/s12887-022-03398-9

**Published:** 2022-06-11

**Authors:** I. Suir, J. Oosterhaven, M. Boonzaaijer, J. Nuysink, M. Jongmans

**Affiliations:** 1 Research Group Lifestyle and Health, Research Centre Healthy and Sustainable Living, HU University of Applied Sciences, Utrecht, The Netherlands; 2 Faculty of Social and Behavioural Sciences, Department of Pedagogical and Educational Sciences, Utrecht University, Utrecht, The Netherlands; 3 Department of Neonatology, University Medical Centre Utrecht, Wilhelmina Children’s Hospital, Utrecht, The Netherlands

**Keywords:** eHealth, Very premature infant, Motor development, AIMS, Neonatal follow-up

## Abstract

**Background:**

In The Netherlands, prematurely born infants and their parents are offered regular developmental check-ups in a hospital setting. In line with providing healthcare at distance, the use of video footage showing the infant’s behavior and movements, taken by parents at home and assessed by professionals online, might be a fruitful future practice. The focus of this study was to gain insight into parental experiences with the Alberta Infant Motor Scale home-video method and their appraisal of its applicability for use in an outpatient neonatal follow-up clinic.

**Method:**

A qualitative descriptive study among parents of healthy extremely or very premature infants (GA 26.2–31.5 weeks) participating in a longitudinal study of motor development between 3–18 months corrected age. Ten semi-structured interviews were conducted and transcribed verbatim. Data was analyzed independently. Inductive content analysis was performed following the process of the AIMS home-video method.

**Results:**

Parents appraised the AIMS home-video method as manageable and fun to do. Instructions, instruction film, and checklists were clear. Transferring the video footage from their phone to their computer and uploading it to the web portal was sometimes time-consuming. Parents gained a better awareness of their infant’s motor development and found the provided feedback a confirmation of what they already thought about their infant’s development and was reassuring that their child was doing well. First-time parents seemed more uncertain and had a greater need for information about (motor) development, but on the other hand, also had confidence in their child.

All parents thought that home-videos can be an addition to follow-up visits, but cannot replace (all) visits. It may be an opportunity to reduce the frequency of hospital visits, while still having their infant monitored.

**Conclusion:**

Parents appraised the AIMS home-video method positively and are of the opinion that home-videos can be of added value in monitoring infants at risk in neonatal follow-up additional to hospital visits. In future research a user-friendly application and/or platform to exchange video footage safely between parents and professionals should be developed with all possible stakeholders involved and implementation should be explored.

**Supplementary Information:**

The online version contains supplementary material available at 10.1186/s12887-022-03398-9.

## Introduction

Early screening and treatment of infants at risk is seen worldwide as an effective way of preventing health and social problems later in life [1–3]. Very premature born infants are infants at risk of developmental disorders, such as problems with gross and fine motor skills, problems with cognition, and social and/or behavioural problems [1, 4–6]. Approximately 30% of these children experience problems with motor skills which often persist throughout childhood and sometimes into adulthood [6]. Early detection of developmental problems is therefore important.

In the Netherlands, between 2017 and 2019, approximately 7% of infants were born prematurely, of which 1.3% were born very or extremely prematurely (< 32 weeks gestational age (GA)) [7]. These infants are admitted to and looked after in hospitals with Neonatal Intensive Care Units (NICU). Because of increases in quality of care, the chances of survival of these infants have increased considerably over the past decades [4]. After discharge, as advised by the European Standards of Care for Newborn Health [8] and according to the protocol from the Dutch Neonatal Follow-Up (LNF) Study Group for infants admitted to the NICU [9, 10], infants and their parents return to the hospital for regular check-ups at the follow-up clinic, where standardized tests are conducted. These tests cover the surveillance of several developmental domains like neuromaturation, motor development, cognitive development, behavioral development, and executive functions. In the Netherlands, the neonatal follow-up (NFU) is provided at the hospital by different care professionals (e.g., neonatalogist, pediatric physiotherapist, pediatric psychologist, language/speech therapist) for examining the development of the infant in the above-mentioned domains at the age of 6, 12, and 24 months CA and at 5 and 9 years [10].

Despite the importance of NFU programs, attendance with follow-up visits decreases over time and there is a need to implement strategies to increase attendance and family engagement in NFU [11].

Using video footage to monitor infants might be a promising supplement to the check-up visits to the hospital. Replacing hospital visits for monitoring motor development by using eHealth technology may reduce costs and may increase efficiency. Besides, recording an infant in its own environment will provide a more realistic image of the infants’ capabilities. Subsequently, it allows multiple professionals to look at the same video repeatedly which may enhance the quality of care [12, 13]. With the internet, it is possible to constantly monitor health conditions, increase sharing of information between parents and care professionals, and with that increase clinical decision making and disease management. In addition, it allows delivering care everywhere and at every time, and with that increases access to care [13, 14]. Even though some studies have shown positive costing outcomes [15, 16], there is still a great need for research providing evidence that eHealth decreases costs.

The need for remote care has become painfully relevant with the COVID-19 pandemic, resulting in many new solutions for providing and continuing care [14, 17–19]. Many digital applications (apps) have been developed for health care purposes in recent years. These apps enable the monitoring of patients, provision of eHealth interventions, and the collection of ‘big data’ [20, 21].

Within the GODIVA-study (Gross mOtor Development of Infants using home-Video with the Alberta Infant Motor Scale), a method has been designed to assess an infant’s motor development in which parents make a video recording of their infant at home, which is then assessed with an observational instrument, the Alberta Infant Motor Scale (AIMS) [22]. For longitudinal measurements of infants for research purposes, repeated filming has already been proved useful and feasible for parents of healthy term-born infants [23, 24]. Because it is often stressful for parents to have prematurely born infants at risk of developmental problems with subsequent need for medical care, the question arises as to whether parents of infants at risk find the AIMS home-video method useful for them as well [25]. In addition, home videos may contribute to monitoring infants at risk. The main purpose of this study was to gain an understanding of parental experiences of infants at risk within the NFU with the AIMS home-video method. Subsequently, parents were asked how they appraised its applicability for use in an outpatient follow-up clinic.

## Method

### Study design

This qualitative descriptive study [26, 27] is part of a longitudinal study, the GODIVA-PIT study (to be reported on later). The GODIVA-PIT study (Gross mOtor Development of Infants using home-Video registration with the AIMS- following Premature Infants in Time) explores the motor trajectories of healthy premature infants (GA ≤ 32.0 weeks and/or with a birth weight < 1500 g) from 3.5 to 17.5 months corrected age (CA). In this study, parents use the AIMS home-video method to record their infant and the footage is assessed on gross motor development.

### Study setting

Participants in the GODIVA-PIT study were recruited between May 2017 and December 2019 at the Wilhelmina Children’s Hospital of the University Medical Centre Utrecht, Radboud University Medical Centre (Nijmegen), Isala Hospital (Zwolle), and by paediatric physical therapists of the TOP programme (Transmural developmental support for VPT infants and their parents) [28] throughout the Netherlands. Infants were recruited at regular neonatal or outpatient follow-up appointments, or during their first contact with the TOP therapist.

### Ethics

The GODIVA-PIT study was approved by the Medical Ethical Board of the University Medical Centre Utrecht (METC/UMCU), with reference number 17–186/C. Parents gave written informed consent prior to participation, in which they also gave consent to be contacted for another related study.

### Sampling

**Fig. 1:**
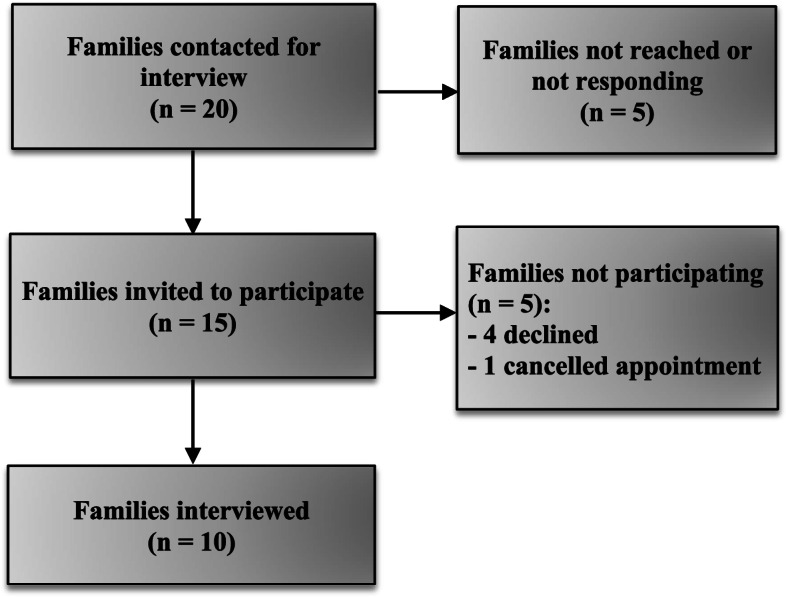
Flowchart for participating families

Via convenience sampling, 20 families participating in the GODIVA-PIT study who had given permission in the Informed Consent to be contacted for other studies were approached, of which 10 agreed to participate (Fig. 1). The interviews were scheduled to commence after the parents had recorded their child at least once. When, after these 10 interviews, data appeared saturated, no further interviews were scheduled.

### AIMS home-video method

**Fig. 2:**
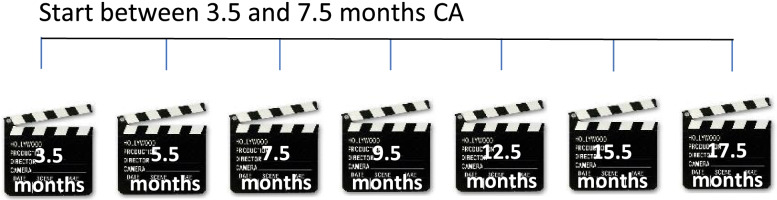
Corrected ages of infants when recorded by their parents

In the GODIVA-PIT study, parents were asked to record their infant with the AIMS home-video method five to seven times starting at the corrected ages of 3.5, 5.5 or 7.5 months until 17.5 months, with intervals of two to three months (Fig. 2). They received three instruction films and a booklet with three corresponding checklists. Parents recorded their infant in their own environment and at their own chosen time. After parents had uploaded the videos via a secure web portal, the researcher and paediatric physical therapist (IS) assessed them with the AIMS. Scoring the AIMS from home-videos has proven to be valid and reliable [22]. Parents were given feedback on their infant’s motor development by email. This email contained objective information on what was seen in the videos, a figure with norm references in which their infant's score was incorporated, and pictograms of the scores on the AIMS (see Additional file 1). Whenever abnormalities were seen in an infant's motor presentation, the attending physician and/or paediatric physiotherapist were contacted for consultation [29].

### Data collection

Between January 2019 and February 2020 face-to-face in-depth semi-structured interviews were conducted. Semi-structured interviewing offers participants sufficient opportunity to express their views and helps to discover information not previously thought of [29]. The interviews were conducted by a pair of interviewers, always consisting of the researcher (IS), who is also a lecturer on the master’s programme Paediatric Physiotherapy, and a student of this programme (CW/AV/AS) who was under the supervision of the researcher. The interviews took place in the family home, with one or both parents present. All interviews were video- and audio recorded.

A guide with a topic list (see Additional file 2) formed the basis for the semi-structured interviews. Interview questions and topics were developed based on the structure of the AIMS home-video method and the previous work of Boonzaaijer et al. [23] and a review of the relevant literature concerning neonatal follow-up and the use of digital tools. A pilot interview was conducted among researchers to test the interview guide. After each interview, deliberation took place with the two interviewers, and the guide evaluated and adjusted when necessary [30]. The guide provided key topics based on the comparative study of Boonzaaijer [23], supplemented with topics regarding parents’ views on using home videos for neonatal follow-up. Feedback on the topic list was provided by two experienced researchers (JN, MJ).

### Data management and analysis

Audio recordings were transcribed verbatim according to a standard protocol. A content analysis approach [31] was used, guided by the research objectives and the model of Boonzaaijer et al. [23]. The phases of open, axial, and selective coding were used for analysis to identify the most relevant themes [32].

Interviews, where both parents were present, were analysed as one interview, with the transcript indicating whether it was the father or the mother who said it. These data were analysed as individual statements, which were given a separate coding, and as such were not parent or interview specific. The software program Atlas.ti was used for analysing and classifying the data [33].

### Dependability and credibility

To enhance the dependability and credibility of the data, all phases of the analysis were performed independently and compared afterward. When no consensus was reached, a third researcher (JN) was consulted. During the first phase of the analysis, the researcher (IS) and two students (CW and KS) performed open and axial coding. In a second phase, all data were analysed by two researchers (IS and JO), including open, axial, and selective coding. During analysis, a journal was kept with reflexive notes. Variation in the population was continuously monitored (i.e., fathers and/or mothers interviewed, infant GA, birth weight, number of times recorded). After nine interviews, data appeared saturated, which the last interview confirmed.

To enhance triangulation, three peer debriefing sessions were held with researchers and physiotherapists working in different fields (neurology, pain, psychosomatics, and paediatrics), a paediatric health psychologist, and a neonatologist. After these sessions, a final peer debriefing session took place to confirm the alterations in choices of quotations and names of the (sub)themes [32].

## Results

We interviewed parents of 10 families: five interviews were conducted with the mother only, two with the father only, and three with both parents.

Mothers’ median age was 34 years (range 28–40), and fathers’ median age was 35 years (range 30–45 years). Eight mothers and seven fathers were highly educated. Infants’ median GA was 29 weeks (range 26.2–37.0), and median birthweight was 1210 g (range 960–2240). Parents filmed on average three times, with a range of one to seven times. One parent was a mother of twins, one parent had a post-migration background, and one infant was suspected of having cerebral palsy during the study. Parent and infant characteristics are shown in Table 1.

**Table 1:** Parental and infant characteristics

Interview	Sex	Parent(s) interviewed	Times recorded	Corrected Age infant at interview	Gravidity	Birth ranking	Health status	Parental country of origin	Parental education (high^a^/ middle^b^/ low^c^)^49^
1	boy	both parents	1	4 month	singleton	first	healthy	Dutch	High / high
2	girl	mother (father came at the end)	2	9 month	singleton	first	healthy	Dutch	High / high
3	girl	father	2	19 month	singleton	third	healthy	Turkish	Middle
4	boy/ boy	mother	3	5 month	twin	first/ second	healthy	Dutch	High
5	boy	mother	3	8 month	singleton	third	healthy	Dutch	High
6	girl	father	2	9 month	singleton	first	healthy	Dutch	High
7	girl	mother	3^d^	13 month	singleton	fourth	healthy	Dutch	Medium
8	boy	mother	4	11 month	singleton	first	healthy	Dutch	High
9	boy	both parents	6^e^	22 month	singleton	first	healthy	Dutch	High / high
10	girl	both parents	7	20 month	singleton	second	suspect of Cerebral Palsy	Dutch	High / high

^a^high education = associate degree programs, higher education, Bachelor programs, Master degree programs, and doctoral degree programs

^b^medium education = upper secondary education, (basic) vocational training, and middle management and specialist education

^c^low education = primary school, prevocational secondary education, and lower secondary vocational training and assistant’s training

^d^parents started participating in a study at the infants age of 5.5 months

^e^parents started participating in a study at the infants age of 7.5 months

The analysis will be presented in two parts, the first relating to the practical aspects of the AIMS home-video method together with the feelings and thoughts of parents using the method, and the second covering the parents’ vision of the use of home videos in neonatal follow-up.

**Fig. 3:**
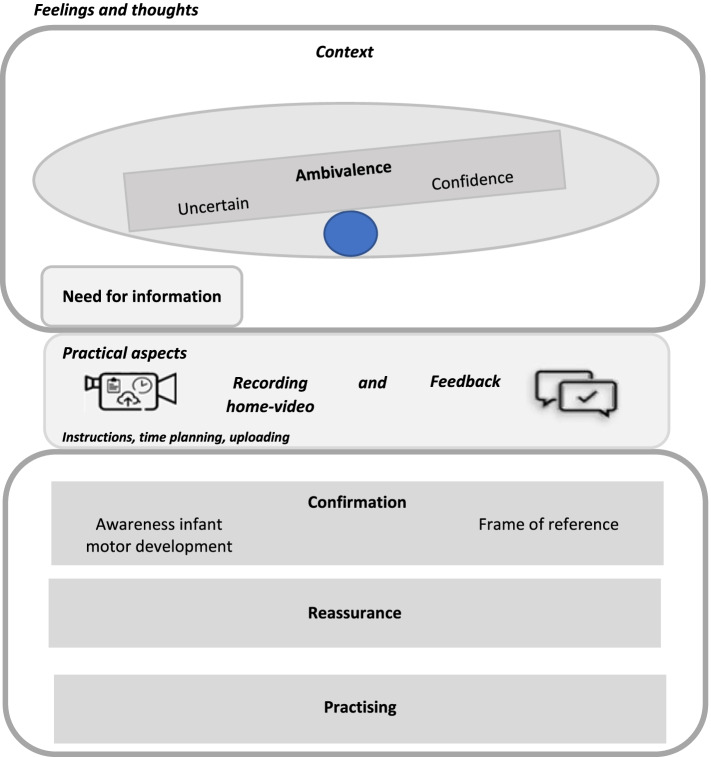
Overview of the themes extracted from the interview data

Figure 3 represents the overview of the practical aspects, and feelings and thoughts about the experiences with the AIMS home-video method. The practical aspects related to the process of making the home video are: the instructions, time planning, recording the video, uploading, and feedback. In Table 2, the extracted themes and subthemes are presented, accompanied by representative quotes.

**Table 2:** Quotations matching the themes and sub-themes regarding the practical aspects, feelings, and thoughts

Theme / *Sub-theme*	Quote
**Instructions** ***Clear*** ***Useful*** ***Exercising***	*M: ‘Yes, those (instructions) were pretty clear. Yes, with that (checklist), you really got* [it]*.’ (int.8)* *F:’I learned … I saw how we had to record this film. So that was also useful.’ (int. 1)* *M: ‘Sometimes we didn't know whether he could perhaps do certain things … or whether not. We thought, oh, perhaps that might be fun to offer* [that activity] *to him now. More like that. Because that’s what we’re going to do.’ (int. 5)*
**Time planning** ***Recording on one day*** ***Two persons*** ***Undressing*** ***The right state*** ***Becomes more easy*** ***Own environment***	*M: ‘You want* [to do] *it in just one day, of course, and that doesn’t always work.’ (int. 3)* *F: ‘Well, the limitation was that both of us had to film it.’ (int. 2)* *M: ‘And filming that while standing, yes, I always need someone for that. And at a hectic pace, it does not always work smoothly.’ (int. 4)* *M: ‘And you have to change his clothes a few times and I did not find that pleasant. He was actually tired by the time I had undressed him.’ (int. 5)* *M: ‘Yes, I usually plan it in my calendar. Then I think, oh, it is a day when we are both there, hey, on the weekend. But then he is just sleeping or then he has just been sick,* [and] *then the moment has passed.’ (int. 3)* *M: ‘It became shorter and shorter, I think, because he could actually do more* [each time] *and it was getting easier.’ (int. 10)* *M: ‘That you don’t again … because you are in the hospital quite a lot. I think that I would not have joined if I had to go somewhere* [to take part]*. I wanted to participate, because it could just be at home.’ (int. 4)*
**Recording home-video** ***Fun*** ***Similar to normal playing*** ***Awareness MD***	*M: ‘I actually found it very nice to do.’ (int. 2)* *M: ‘You actually film what you already do with him every day.’ (int. 4)* *F: ‘It’s nice that you see those different videos, that* [motor] *development. Then you are much more aware, I think. Otherwise you are not so aware of it day to day.’ (int. 5)*
**Uploading** ***Time-consuming***	*M: ‘It really takes one or two hours (with transferring and uploading). So that is tough.’ (int. 3)*
**Feedback** ***Frame of reference***	*M: ‘Especially with regard to how he is developing compared to other children of his age, corrected and not corrected [*for his prematurity*]. That's actually what I like most about it. And that you sort of look at how is he on the curve, is he going this way or that way. But above all, does it fall within the normal* [range]*?’ (int. 3)*
**Context** ***Need for information*** ***Uncertain*** ***Reassurance*** ***Ambivalence*** ***Confidence*** ***Confirmation***	*M: ‘And then I notice that I think, hmm, is that the way it should be? Or should he actually be able to* [do that]*? Or what is in it?’ (int. 7)* *M: ‘But sometimes I find that difficult, because I don’t… because I sometimes get insecure, because they are born too early.’ (int. 7)* *F: ‘What else can go wrong, that was the hardest, I think. That matters a lot, in that it is nice to see again … that we get confirmation that it's going well, orally, on paper and on screen.’ (int. 9)* *M: ‘I am very confident that I want my children, I want to stimulate them in their development, if that is necessary, but I also want them to actually do their own thing. Should I encourage them more because it's good for them, or should I let them do it themselves?’ (int. 7)* *M: ‘He* [was] *just born too early. …so* [there’s] *no reason why he shouldn’t reach his milestones.’ (int. 3)* *M: ‘It is a kind of confirmation of what you actually feel yourself.’ (int. 6)*

*M* = Mother

*F* = Father

### Part I: Parental experiences with the AIMS home-video method

#### Instructions

All parents considered the instructions on the checklists in the booklet ***clear***. Most parents watched the first instructional video on how they could film their infant, which was regarded as ***useful***. But it was not always clear that the three checklists entirely corresponded to the instruction videos. As a result of the instructions on the checklists, some parents actually started ***practising*** some of the items with their infant.

#### Time planning

This was the most challenging part of the home-video method. ***Recording on one day***, the necessity for ***two persons*** to record when the infant was young, ***undressing*** the infant, and having the infant in ***the right state***, were perceived as the most bothersome for recording. But parents also reported that recording ***became easier in time****,* since:1) parents knew what to expect from recording; 2) the infant slept less, so planning became easier; 3) the urge for two people to film was reduced, due to improvement of the motor abilities of the infant.

Recording their infant in its ***own environment*** and choosing the right moment was appreciated and sometimes a prerequisite, or even the decisive factor, for participating in the study.

#### Recording home videos

Most parents experienced recording their infant as ***fun*** to do. Some parents said that prematurity made them more careful about handling their infant, when it was very young. Other parents mentioned that handling their infant for the video was ***similar to normal playing***. But if the infant was comfortable at the moment of recording, positioning the infant was easy.

During recording, parents occasionally discovered new motor skills in their infant, gaining a better ***awareness*** of their infant’s motor development. In one interview with both parents, they said that because of the different recordings, one could actually see the development. Additionally, it made them more aware of what their infant already did than they would usually be during normal days.

#### Uploading

Most parents did not report any problems uploading the films to the web portal, although sometimes it was perceived as ***time-consuming***. However, some parents struggled with transferring the videos from their telephone to the computer. Suggestions for making uploading easier concerned mainly the user-friendliness of the web portal, e.g. by using an application on one’s mobile phone.

#### Feedback

The feedback parents received was in general considered clear and valuable. The figure in the feedback (Additional file 1) provided a ***frame of reference*** in which parents could see how their infant was developing, compared to peers*.* Interpretation of the graph with the norm references of term-born infants and premature infants was sometimes challenging, though the text below the figure and the explanation of the results in the email clarified this.

Generally, the feedback provided was a ***confirmation*** of what parents already thought about their child's development and, further, gave ***reassurance*** that their child was doing well. One father said that, while he knew what might go wrong in development due to the prematurity of his child, when he heard and saw that his child was doing well, he felt reassured. Besides, according to some parents, it was pleasant to have an extra pair of eyes monitoring their infant.

### Context

Parents expressed the view that having a premature infant is stressful, with the realisation of having a different start with their infant than expected. The context of either being a first-time parent or already having more parenting experience also seems to matter.

Even at the time of admittance to the NICU, some parents had questions about their infant’s development and felt the ***need for information.*** Later, parents also had questions about what their child should be able to do at certain ages, and whether their child’s actual repertoire was appropriate to their age. First-time parents seemed more ***uncertain***, reflected in feelings of doubt about their infant’s development and hence a greater need for information about (motor) development. Recording their child made their infant's newly acquired motor abilities obvious, and feedback was found ***reassuring***. A few parents conveyed the impression of being inspired to practice with their child, according to the instructions. Although these parents created the impression of being more uncertain, some ***ambivalence*** emerged in that they also had ***confidence*** in their child. The received feedback was often considered a ***confirmation*** of what they already thought about their child.

Parents who already had parenting experience seemed less uncertain and more confident about their infant’s development, reflected in having more faith in their infant's own pace in motor development. They reported less need for information and did not mention noticing new motor abilities, but expressed the need for comparison with their infant’s peers and for confirmation of what they already thought (i.e., that their child was doing well). Also, experienced parents did not mention practising with their child prompted by the instructions and/or recording.

#### Atypical motor development

The parents of the infant thought to have cerebral palsy reported similar themes despite differences in the ***context*** where their child showed atypical motor development during the study.

These parents also became more ***aware*** and gained more knowledge about their infant’s motor development through ***recording*** their child and receiving ***feedback***. As a result of this feedback, they could see for themselves that their child was diverging from the norm. This divergence reinforced the concern that their child was not developing as expected and was also a ***confirmation*** of what the doctor had said.

Because of the recording and feedback, the parents of the infant with the atypical motor development reported noticing more about what their child could do, rather than what she or he could not do or should be able to do, according to standards. This may also be interpreted as ***reassuring***. Also, they were searching for a ***frame of reference*** for themselves, because the comparison with their older child was no longer valid.

### Part II: Use of home videos for neonatal follow-up

Parents uniformly agreed that using home videos for monitoring infant motor development can certainly be an ***addition*** to follow-up visits but should ***not*** be a ***substitute*** for these. For instance, video recordings could be used in addition to regular check-ups when the doctor or parents themselves have questions about progress in other developmental domains, e.g., language or communication. In addition, parents consider using video footage as a way of providing information to other involved professionals, such as doctors in other hospitals or speech therapists. Also, some parents considered the use of home videos as an opportunity to reduce the frequency of hospital visits, while still having their infant monitored.

On the other hand, parents emphasised the importance of doctors discussing with parents in person whether they wished to film their child: the importance and benefits of recording have to be clear at all times. Also, clear instructions, such as provided in the current study, should be given to parents on how and what to film.

## Discussion

The present study describes the practical experiences, feelings, and thoughts of parents of very preterm infants with the AIMS home-video method. In addition, parents gave their views on the suitability of home videos for use in outpatient follow-up clinics. Overall, parents found the AIMS home-video method to be manageable and fun to use, especially as infants get older; only transferring recordings from their phone to the computer and uploading them to the web portal was experienced as time-consuming. Parents gained a better awareness of their infant’s motor development and found the feedback to be reassuring, confirming that their child was doing well. All parents are of the opinion that home videos can be a useful addition, but not a replacement for, follow-up visits.

The GODIVA-PIT study was conducted in a similar Dutch (health care and cultural) context and used the same methodology as in the study of Boonzaaijer et al. [23] The main difference between the studies concerned the birth status of the children is that the current study included parents of preterm infants, where the study of Boonzaaijer et al. included parents of term-born infants. The majority of the (sub)themes in practical aspects and feelings and thoughts emerged in both studies, with only the content of the (sub)themes being different. In practical aspects, few differences arose, which may be explained by the improved digital capabilities of the mobile phones nowadays and the better functioning web portal (learning from previous errors). For instance, parents of premature infants did not experience digital errors in uploading videos and low capacity for storage of footage on their mobile phones, unlike the parents of the term-born infants. Nevertheless, in both studies it often took a long time to upload the videos [23]. The major differences with the study of Boonzaaijer et al. are in the content of the (sub)themes of the feelings and thoughts, formed by the difference in the journey they have had in the birth of their premature infant. Parents of premature infants often experience a sudden disruption of the pregnancy, which makes them parents sooner than expected [34]. Next to this unexpected birth, the medical care is longer and more intensively accompanied by insecurities about their infant’s wellbeing and future expectations than with healthy term-born infants [35]. When combined with becoming a parent for the first time, it seems natural to have feelings of uncertainty and to need information. That this uncertainty and need for information is less for parents of more than one child may be attributed to learning from experience, where parents use their experiences with their firstborns when faced with similar situations with subsequent children [36, 37]. Experiences acquired with their firstborns increase their knowledge and effectiveness in meeting the needs and demands of later-born children [24, 38]. Parents appear to feel uncertain and vulnerable when they lack information on how to enhance their child’s care [39]. In response to this uncertainty, it seems natural that parents of premature infants express their need for reassurance and confirmation that their child is doing well and that they are doing the right thing [35].

Interestingly, parental beliefs seem to play a role in expectations of development [40, 41]. In our study, first-time parents felt that they should actively stimulate their child's motor development, while experienced parents were happy to trust their infant's own pace.

According to published research, parents in different cultures also differ in their beliefs about their infants’ motor development and may therefore show differences in parental practices. For instance, first-time Israeli mothers of term-born infants attributed a bigger role to stimulation, whereas Dutch first-time parents attributed a bigger role to maturation and infants’ own pace in development [41].

This study also gives insights into the appraisal of home videos for monitoring infants. Actively involving parents in neonatal follow-up perhaps contributes to Family Centred Care (FCC), which is supposed to enhance (motor) outcomes of premature infants [42]. This is also seen in the transition from the NICU stay to the family’s home, where FCC principles and interprofessional collaboration promote the well-being of the family by enhancing parents’ autonomy and self-confidence [43]. In our study, recordings made parents aware of their infant’s motor development, which may enhance empowerment and allows for increased confidence in parenting [44, 45]. Giving feedback reassured parents and confirmed how their child was doing, which may decrease stress levels in parents. These factors, empowerment and decreased stress, may contribute to the (motor) development of the infant [24, 42, 44].

A relevant lesson learned from this study is that, when giving feedback, it is very important to tell parents what their child *can* do, as the parents of the infant with suspected cerebral palsy stated. It is important to concentrate on the strengths of a child, with positively phrased messages, and not just focus on weaknesses [35, 36, 46, 47]. This is in agreement with the strength-based principle, whereas research shows that positive communication enhances parents’ confidence and reduces their anxiety. Negative communication effects reported are difficulties for parents in adapting to, and accepting their infants’ health [46].

### Strengths and limitations

Some limitations and strengths can be identified concerning the quality of the study. First, there was no member check to confirm whether the interpretation of the results as presented here was recognizable, which would have contributed to the credibility of the data. Second, a convenience sample was used, which is more of a risk compared with a purposive sample. However, there appeared to be a sufficient reflection of the sample in the parent (fathers and/or mothers interviewed), infant (GA, birthweight), and study characteristics (number of times recorded and therefore age of the infant during recording). On the other hand, there was only one infant with atypical motor development. Parents gave different information, although almost all themes emerged in these interviews, though with different content. A further point is that most parents were highly educated [48]: research among Australian parents on the use of an application to assess infant general movements captured on a video made by parents showed that, while most parents used the Babymoves app successfully, parents of lower socio-demographic status used the app less [49]. Lastly, as in all research, the only parents participating were those interested in the study, which raises questions about whether the AIMS home-video method is usable for monitoring all infants.

To increase rigor of the interpretation of the data, the researchers endeavoured to be reflexive in the iterative process by making notes during the process and by independent coding. Arranging critical peer feedback and peer debriefing sessions where different perspectives on the data were involved enhanced triangulation.

### Future research

Following the studies on parents' experiences of healthy term and preterm infants with the AIMS home-video method [23], it seems important for future research to actually implement a home-video method. The implementation should involve research into parent preferences and adaptations during an iterative implementation process within the neonatal follow-up system. The experiences of parents, as well as of the professionals involved, in using such a method as part of their clinical practice, should be explored. Practical implications of the implementation (e.g., when will parents be asked to make a video, who will ask parents, and who will watch and score the video) in the care-process need to be mapped. Besides, the method can also be used by other health care professionals who are trained in scoring and interpreting the AIMS test results. To enable implementation, the already available knowledge with the current web portal and knowledge gained in the GODIVA research projects [22–24] should be used to further develop a user-friendly application or platform to exchange video footage safely. For neonatal follow-up, such a platform should preferably be integrated into hospital management software. During development, it is important to involve parents of different (post-)migration backgrounds, and education levels, including parents of infants with atypical motor development. Finally, it will also be important to involve stakeholders like health insurance companies to ensure it will be part of the insured care.

## Conclusion

Parents of preterm infants find the AIMS home-video method to be manageable while receiving feedback reassures them and confirms that their child is doing well. Moreover, this method appears to be an intervention that enhances the empowerment of parents in providing insight into their infant’s motor development. It is suggested that home videos can be of added value in monitoring infants at risk in neonatal follow-up in addition to hospital visits and to inform many of the health care professionals involved.

In future research, a user-friendly application and/or platform to exchange video footage safely should be developed with all possible stakeholders involved and implementation should be explored.

## Supplementary Information

**Additional file 1.** Example of the feedback to parents after an assessment.

**Additional file 2.** Interview guide.

## Data Availability

The data generated during and analysed during the current study are not publicly available due to the qualitative nature of the study and thereby sensitivity of the data. Data are available from the corresponding author on reasonable request. Data of the GODIVA-PIT study are stored in the repository of the Utrecht University of Applied Sciences.
