# Supplementary material for: The AIMS home-video method: parental experiences and appraisal for use in neonatal follow-up clinics
Source: BMC Pediatr. 2022 Jun 11;22:338. doi: 10.1186/s12887-022-03398-9 (PMC9187888; doi:10.1186/s12887-022-03398-9)
Supplement: Supplementary file 1 — Additional file 1. Example of the feedback to parents after an assessment. [file 12887_2022_3398_MOESM1_ESM.docx]

**Additional file 1: Example of the feedback to parents after an assessment.**

**NAME**

**… month (corrected age)**

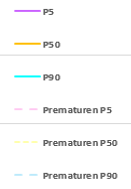

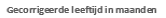

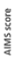


AIMS score

5^th^ centile rank

50^th^ centile rank

90^th^ centile rank

Premature 5^th^ centile rank

Premature 50^th^ centile rank

Premature 90^th^ centile rank

Corrected age in months

|  | **Explanation 5^th^, 50^th^ and 90^th^ centile rank**  In the graph above, your child's score is represented in relation to the 5**^th^**, 50**^th^** and 90**^th^** centile rank.  A centile score of 50 indicates that a child shows average motor development compared to peers.  This score is a moment in time. It is an indication of where the child is in its motor development compared to peers at that time.  At the corrected age of x month, NAME falls between the 25th and 50th centile rank in respect to children born on time.  Compared to children born prematurely he/she falls between the 75th and 90th centile rank. This means that he/she scores according to  the standards of motor development in comparison with peers who were also born prematurely. | | | | | | | | | |
| --- | --- | --- | --- | --- | --- | --- | --- | --- | --- | --- |
|  | |  |  |  |  |  |  |  |  |  |
|  | |  | | | | | | | |  |


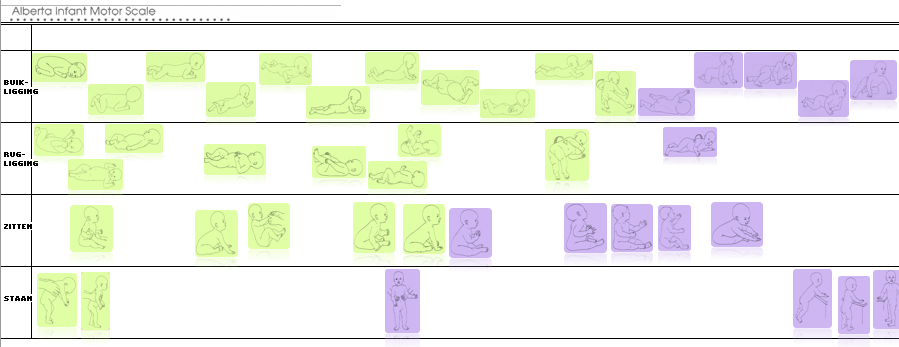


Stand

Supine

Sit

Prone

|  | **Explanation**  Above you can see the score form of the instrument (AIMS: Alberta Infant Motor Scale) that charts motor development.  Based on the recordings of your child, we assessed the score and coloured the pictures which represent the items we score.  The green pictures display the items your child has shown in the video or has already mastered. The purple pictures are items your child  did not show during the video.  The pictures are shown in chronological order of motor development. Because every child follows his/her own development, it is  possible that your child skips an item.  To score a picture, specific requirements are set for the performance and posture. It is therefore possible that you think your child has  shown a posture/movement which we have not been allowed to score, because the performance does not meet the requirements.  **Translated with www.DeepL.com/Translator (free version)** | | | | | | | | | |
| --- | --- | --- | --- | --- | --- | --- | --- | --- | --- | --- |
|  | |  |  |  |  |  |  |  |  |  |
|  | |  | | | | | | | |  |

**NAME**

**x month (corrected age)**
