# Supplementary material for: The AIMS home-video method: parental experiences and appraisal for use in neonatal follow-up clinics
Source: BMC Pediatr. 2022 Jun 11;22:338. doi: 10.1186/s12887-022-03398-9 (PMC9187888; doi:10.1186/s12887-022-03398-9)
Supplement: Supplementary file 2 — Additional file 2. Interview guide. [file 12887_2022_3398_MOESM2_ESM.docx]

**Additional file 2: Interview guide.**

| **Topic list** | | |
| --- | --- | --- |
| **Start** | | |
| - Thank you for your cooperation in this interview  - Explanation of what is going to happen:  “The purpose of the interview is to gain insight into the experiences of parents with the home video method. This makes it possible for us to improve and adjust the method. The interview is about your personal experiences with the GODIVA-PIT method. We would like to learn from your experiences. We are also interested in knowing how parents think about using videos made by parents, for doctor visits, like the neonatal follow-up visit, where you are going to with your child.  - Explaining some practical things: duration (about 45-60 minutes), recording equipment, anonymity)  - There are no right or wrong answers! What matters to us is your opinion. We want to learn from your experience. | | |
| **Topic** | **Question** | **Extra questions** |
| **Start** | How did you like recording your child for this study? | - facilitators and barriers |
| **Time Planning** | Can you tell us the process of recording?  Approximately how much time do you think you spent recording? | - organising/finding the right moment  - positive/negative aspects of recording at home  - time investment (every time, frequency, age of child) |
| **Video footage (technically)** | What did you think of all the instructions? | - watching instructional videos  - read the instruction booklet  - checklists  - clear  - findability |
| **Video footage (elicitation movement)** | What did you think of eliciting your child?  How did you experience your participation in the study? | - performance child  - what you yourself have gained  - insight motor skills |
| **Uploading** | What did you think about the uploading of the videos? | - manual/instructions for uploading  - uploading difficulties/problems  - duration  - expectation in advance  - app? |
| **Feedback** | What did you think of the feedback you got on the video footage? | - understanding feedback  - influence feedback on handling |
| **Follow up** | Do you think the video method is suitable for parents visiting the neonatal follow-up?  Why yes/no?  How do you envision using the video method for the neonatal follow-up? | - facilitators and barriers  - added value |
| **Prematurity** | What do you think of the video method for children born prematurely?  Can the method also be of added value for other children (with or without problems)? | - confronting  - expectations own child |
| **Expectations** | | |
|  | How stressful did you find recording your child for your child and yourself?  In the meantime, have you considered stopping the study? If so, why?  What motivated you to continue the study? |  |
| **Reflection** | | |
|  | What was your opinion about recording beforehand?  Was recording as expected? Why or why not?  What would you do differently next time?  What do you think could contribute that fewer parents drop out?  Do you have any questions about privacy?  Do you have anything to add to this interview? |  |
